# Human Babesiosis in Europe

**DOI:** 10.3390/pathogens10091165

**Published:** 2021-09-09

**Authors:** Anke Hildebrandt, Annetta Zintl, Estrella Montero, Klaus-Peter Hunfeld, Jeremy Gray

**Affiliations:** 1 St. Vincenz Hospital Datteln, Department of Internal Medicine I, 45711 Datteln, Germany; a.hildebrandt@vincenz-datteln.de; 2 Institute of Medical Microbiology, University Hospital Münster, 48149 Münster, Germany; 3 UCD School of Veterinary Sciences, University College Dublin, D04 W6F6 Dublin, Ireland; annetta.zintl@ucd.ie; 4 Parasitology Reference and Research Laboratory, Centro Nacional de Microbiología, Instituto de Salud Carlos III, Majadahonda, 28220 Madrid, Spain; estrella.montero@isciii.es; 5 Institute of Laboratory Medicine, Microbiology & Infection Control, Northwest Medical Center, Medical Faculty Goethe University Frankfurt, Steinbacher Hohl 2-26, 60488 Frankfurt am Main, Germany; K.Hunfeld@em.uni-frankfurt.de; 6 Society for Promoting Quality Assurance in Medical Laboratories (INSTAND, e.v.), Ubierstraße 20, 40223 Düsseldorf, Germany; 7 ESGBOR Study Group of the European Society for Clinical Microbiology & Infectious Diseases (ESCMID), ESCMID Executive Office, P.O. Box 214, 4010 Basel, Switzerland; 8 UCD School of Biology and Environmental Science, University College Dublin, D04 N2E5 Dublin, Ireland

**Keywords:** European babesiosis, *Babesia divergens*, *Babesia venatorum*, *Babesia microti*, *Ixodes ricinus*, parasite identity, epidemiology, clinical cases, diagnosis, treatment

## Abstract

Babesiosis is attracting increasing attention as a worldwide emerging zoonosis. The first case of human babesiosis in Europe was described in the late 1950s and since then more than 60 cases have been reported in Europe. While the disease is relatively rare in Europe, it is significant because the majority of cases present as life-threatening fulminant infections, mainly in immunocompromised patients. Although appearing clinically similar to human babesiosis elsewhere, particularly in the USA, most European forms of the disease are distinct entities, especially concerning epidemiology, human susceptibility to infection and clinical management. This paper describes the history of the disease and reviews all published cases that have occurred in Europe with regard to the identity and genetic characteristics of the etiological agents, pathogenesis, aspects of epidemiology including the eco-epidemiology of the vectors, the clinical courses of infection, diagnostic tools and clinical management and treatment.

## 1. History

The first reported case of human babesiosis in Europe, and indeed in the world, occurred in 1956 in the former Yugoslavia, now Croatia, in a 33-year-old tailor and part-time farmer who had been splenectomized following a traffic accident 11 years earlier [1]. He presented with fever and severe hemoglobinuria eight days after first feeling unwell and died two days later. The parasites detected in blood smears were identified as *Babesia bovis.* However, *B. bovis* is not known to be zoonotic, and the photomicrographs in the published case report show divergent piroplasms that are characteristic of *Babesia divergens*, as well as a cattle parasite and first described by M’Fadyean and Stockman in 1911 [2]. The second recorded case, another *B. divergens* infection, which also ended fatally, occurred in 1967 in a splenectomized man who had apparently contracted the infection on holiday in the west of Ireland [3]. Further cases then followed in the 1970s in the UK and France, and to date, cases have been recorded in at least 19 European countries, almost always fulminant in splenectomized patients and attributed to *B. divergens*.

A second zoonotic species emerged in 2003 in Italy and Austria [4], initially designated EU1, but now named *Babesia venatorum*. To date, infections with *B. venatorum* have been reported from Germany [5], Austria [6] and Sweden [7], all in splenectomized patients who survived, which possibly indicates a milder course of infection than *B. divergens*, though treatment has improved markedly since the first appearance of zoonotic babesiosis.

The most recent addition to the list of autochthonous zoonotic European *Babesia* spp. is *Babesia microti*, the first confirmed case of which occurred in Germany [8], and caused moderate illness in a spleen-intact but immunocompromised patient. A few mild or asymptomatic other cases have since been recorded, but it is very clear that the strains of *B. microti* present in Europe, where it is common in rodents and ticks, are not as infectious or pathogenic to humans as those in the USA, where *B. microti* infections give rise to approximately 2000 zoonotic babesiosis cases annually [9].

## 2. Parasite Identity

**Figure 1:**
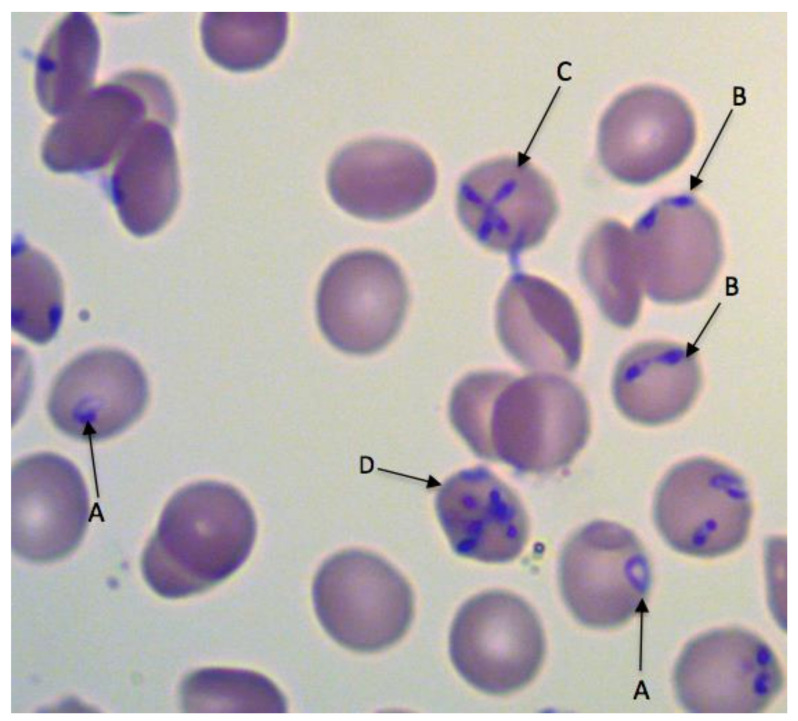
*Babesia divergens* in a Giemsa-stained thin blood smear. Round (**A**), paired pyriform (**B**), tetrad (Maltese cross) (**C**) and multiple parasite (**D**) forms are indicated. Similar round and paired pyriform forms have been observed in infections of *B. venatorum,* and round and tetrad forms occur in *B. microti* infections. Multiple parasite-infected erythrocytes are often seen in high parasitemias. © Estrella Montero, Luis Miguel Gonzalez.

Two of the three *Babesia* species that infect humans in Europe, *B. divergens* and *B. venatorum,* belong to the *Babesia* sensu stricto (s.s.) group and are closely related, (Clade X; [10]), while the third species, *B. microti*, is phylogenetically distinct, belonging to *Babesia* sensu lato (s.l.) (Clade I; [10]). The three parasites are distinguishable morphologically in Giemsa-stained blood smears, but only by experienced diagnostic microscopists because they share important features. For example, divergent paired pyriforms are characteristic of both *B. divergens* and *B. venatorum*, while pyriform tetrads occur in both *B. divergens* and *B. microti.* The much more frequently observed single round trophozoite (‘ring’ stage) occurs in infections of all three species (Figure 1).

*Babesia microti* can be distinguished serologically from *B. divergens* and *B. venatorum*, but the latter two are antigenically similar [4]. Serology is further limited by the time required for an antibody response to develop, which may be several weeks in immunocompromised patients [5]. DNA sequence discrimination is not only relatively swift, but can also be used to identify *Babesia* species, and it is now the method of choice for determining parasite identity. The 18S rRNA gene is by far the most commonly used locus for *Babesia* identification. There are several well-established and sensitive nested PCR protocols targeting this gene, as it is easy to amplify and there is much sequence information in the GenBank database.

**Figure 2:**
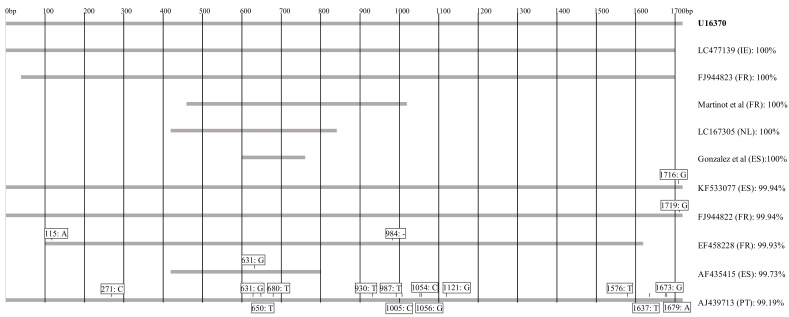
The relative length, positions and heterologies of 18S rRNA sequences of *Babesia* species isolates from human cases compared to the reference sequence U16370. Numbers refer to positions in the reference sequence. Identity scores are according to Clustal Omega. ‘-’ indicates that the base was missing.

It has long been accepted that cattle are the main, if not only, reservoir hosts for human *B. divergens* infections in Europe and sequencing of the 18S rRNA gene strongly supports this. Additionally, 5 of the 10 human *B. divergens* isolates for which sequence information is available are 100% identical with bovine isolate GenBank: U16370 (Figure 2), widely used as a reference sequence for *B. divergens* [11]. Another three isolates showed more than 99.9% homology with this bovine isolate reference, but a further two human isolates probably did not have a bovine origin, showing homologies of only 99.7% (GenBank: AF435415) and 99.2% (GenBank: AJ439713). The first of these cases occurred in a 34-year-old asplenic male Canary Island resident with typical clinical signs of acute babesiosis [12]. The patient reported having removed several unidentified ticks two weeks prior to being admitted to hospital. Since *I. ricinus* is apparently absent in the Canary Islands and the patient had no history of travel, the authors suggested the protozoan may have been transmitted by *Ixodes ventalloi*, an endophilic tick species that is common in the islands and primarily infects lagomorphs, carnivores and rodents [13]. However, this tick has never been confirmed as a vector for *B. divergens* or any other zoonotic *Babesia* species. The second case was reported in a 66-year-old acutely ill splenectomized patient in Portugal [14]. As the patient had travelled to Florida, the USA and the UK six weeks prior to his illness, it was not possible to ascertain where he had been infected.

There are several reports of the detection of *B. divergens* DNA in red deer (*Cervus elaphus*), roe deer (*Capreolus capreolus*) and reindeer (*Rangifer tarandus*) [15], and it has been suggested that these host species, especially red and roe deer, may serve as a source for infection for both humans and cattle. However, none of the deer isolates show 18S rRNA homologies of more than 99.9% with the bovine reference strain, and so far there is no evidence that deer ‘*Babesia divergens*’ have ever caused either human or bovine babesiosis.

It is important to point out, that although useful for diagnostics, the 18S rRNA gene probably cannot unequivocally distinguish between *Babesia* species or strains. The reason for this is its highly conserved nature across the genus, on the one hand, combined with considerable intraspecific sequence diversity, on the other hand, particularly for *B. divergens* and *B. microti*. The cytochrome c oxidase subunit I (COI) gene locus has greater genetic diversity than the 18S rRNA gene and is therefore a more useful tool to distinguish between different species but, unfortunately, it has a lower amplification efficiency [16]. However, when examining the role of potential reservoir species, attempts should be made where possible to sequence sizeable fragments of both the COI and 18S rRNA genes [17]. Other loci such as the beta-tubulin, heat shock protein and merozoite surface protein genes have been used to distinguish *Babesia* isolates; however, until more information for these loci is available in the database, they are unlikely to be used more widely.

The clinical presentation of *B. venatorum* infections can closely resemble that of *B. divergens* [4], but this species is clearly distinct, showing only 98.2% homology with the bovine *B. divergens* reference sequence, U16370. Of the six infections reported in Europe so far, four 18S rRNA sequences are available, including the original two with identical sequences GenBank AY046575 [4], one showing 99.7% [5] and one showing 99.6% (asymptomatic–GenBank: KP072001) homology with AY046575. Almost all 18S rRNA sequences of *B. venatorum* from roe deer (*C. capreolus*) are identical to the first human isolates, with the few exceptions differing by one or two nucleotides, and there can be little doubt that roe deer is the main reservoir host of *B. venatorum* in Europe. It is also interesting to note that *B. venatorum* is not restricted to Europe and approximately 50 cases have been diagnosed in China [18], with the available 18S rRNA sequences being identical to the original European isolates or differing by only one or two nucleotides. The suspected but unconfirmed vector and reservoir hosts are *Ixodes persulcatus* and sika deer, or *Cervus nippon*, respectively [19].

*Babesia microti* is considered to be a species complex, mainly infecting small mammals. Goethert and Telford [20] assigned the parasites in the group to three clades based on analysis of the 18S rRNA and beta tubulin genes, with most of the zoonotic genotypes within Clade 1, which also includes the ‘U.S. genotype’ (e.g., GenBank: AY693840), responsible for the vast majority of human babesiosis cases. Human *B. microti* infections have expanded across the northeast of the USA over the last few decades [21], and several cases in Europe have been associated with travel from that country. In contrast, very few autochthonous cases have occurred in Europe, the first authenticated one was in Germany in an immunocompromised patient and caused by a strain (Jena–GenBank: EF413181) closely related to the USA genotype [8]. Welc-Faleciak et al. reported the detection of DNA of the same genotype in two other individuals, both asymptomatic, who were participating in a survey of forest workers in Poland [22]. Another *B. microti* strain, the ‘Munich’ type (GenBank: AB071177) is widely distributed in Europe and was originally presumed to be non-zoonotic [23]. However, DNA of this strain has reportedly been detected in seven patients in Europe, six of whom presented with various symptoms following a tick-bite in Poland (GenBank: KT429729; [24]), and one who presented with non-specific symptoms in Spain (GenBank: KT271759; [25]). A 157 nucleotide DNA fragment of the Munich strain was detected in an eighth patient, originally thought to be suffering from a prolonged bout of malaria while living in Equatorial Guinea. However, the patient made several visits to Spain during that period [26], and it is difficult to determine whether this *B. microti* infection, successfully treated with antibabesials, was contracted in Spain or Africa. All patients infected with the Munich strain were immunocompetent and only this latter patient had detectable parasites (at a very low level) in thin blood smears. These isolated reports indicate that two European genotypes of *B. microti* can infect humans, but that they are considerably less pathogenic than those in the USA. All cases that were imported into Europe appear to have an American origin (mainly North America), and although only three 18S rRNA sequences are available at present (showing 100% identity to the original American isolate GenBank: AY693840), it is probable that they were all caused by parasites closely related to this widespread U.S. genotype.

## 3. Pathogenesis

Most what is known to date on babesiosis pathobiology has resulted from in vitro experiments and animal studies (mainly in mice and cattle) on *B. microti*, *B. bovis* and *B. divergens* [27,28,29,30,31]. In many human infections, no isolates have been obtained for further investigations, and little information is available on the pathogenesis of *B. venatorum*. Babesia parasites occur within erythrocytes and as extracellular forms in the blood. They multiply within the erythrocytes by a form of budding to produce two or occasionally four daughter cells (merozoites). In fulminant human infections and in highly infected in vitro cultures, multiple parasites may occur within individual erythrocytes (Figure 1). The release of merozoites and eventual erythrocyte lysis is associated with fever and other clinical symptoms including hemolytic anemia, jaundice, hemoglobinuria, obstruction of renal arterioles and renal failure [32]. In vitro observations suggest that erythrocytes are not necessarily completely destroyed when the parasites leave them, but they are damaged. Their optical density decreases, they are reduced in size and are probably removed by the spleen soon afterwards [27,28,29,30,31]. While intermittent episodes of fever have been reported in cases of human babesiosis [32], they typically do not have the same regularity as febrile episodes in malaria, probably because of the asynchronous nature of babesia multiplication and egress from the erythrocytes [33]. In addition to erythrocyte lysis and metabolic alterations, excessive proinflammatory cytokine production contributes to clinical complications [34,35], potentially resulting in vascular leakage, adult respiratory distress syndrome, hypotension and shock [35,36].

Both innate and adaptive immune mechanisms limit the severity of babesial infection [34,37,38]. The spleen plays a central role in host defense by clearing infected erythrocytes from the bloodstream and mounting the protective immune response. The heavily vascularized organ consists of red-pulp and white-pulp zones surrounded by a trabecula and an outer capsule. The marginal zone contains macrophages and neutrophils that recognize and ingest babesia-infected erythrocytes and circulating free parasites as the blood travels through the spleen (in humans, erythrocytes pass through the spleen approximately every 20 min [39]). The red-pulp infected erythrocytes are captured in sieve-like slits in the sinuses as they return to the main circulation and are ingested by macrophages [40,41]. The white pulp of the spleen contains T-cells that produce cytokines, for example gamma interferon (IFNγ), which activate macrophages to phagocytose and destroy parasites, as well as B-cells to secrete babesia-specific antibodies [41]. Antibodies neutralize pathogens, thereby preventing them from entering erythrocytes, and also enhance phagocytosis by macrophages and neutrophils through opsonization and eradicate pathogens through antibody-dependent cytotoxicity by natural killer cells and through the activation of complements [41]. The importance of cellular immunity in controlling parasitemia is demonstrated by the fact that both laboratory mice and humans with depressed cellular immunity have difficulties in controlling infections [5,28,31]. Similarly, the depletion of host macrophages and natural killer cells in mice increases susceptibility to infection [30], while an impaired antibody response due to hematological malignancies and/or rituximab therapy can also lead to difficulties in clearing infection in humans, despite adequate antibabesial therapy [5,42].

In general, factors responsible for severe infections following splenectomy include the delayed and impaired production of immunoglobulin and lack of splenic macrophages, resulting in a reduction in the numbers of infected erythrocytes removed [43]. Consequently, asplenia or hyposplenism often results in fulminant illness and death [1,44,45,46,47].

Babesia parasites possess a number of evasive measures to avoid immune attack, which can lead to persistent infections, even in the presence of an intact immune system [41]. Persistent infections (often asymptomatic) are particularly evident in *B. microti* infections of humans, but less so in *B. divergens* and *B. venatorum* infections, which are usually acute, although infection of their natural hosts (cattle and roe deer, respectively) tend to be persistent. The mechanisms for immunoevasion are unclear, although antigenic variation probably occurs to some extent. Capillary sequestration of infected erythrocytes, thus avoiding circulation through the spleen, has only been reported for certain non-zoonotic species (*B. bovis* and *B. canis*) [48].

## 4. Vector Biology

All zoonotic *Babesia* spp. in Europe are transmitted by the castor bean tick, *Ixodes ricinus.* This three-host tick species spends most of its life (>98%) free living, either host seeking or developing to the next stage. It requires a high humidity at the base of the vegetation (RH >80%), and ideal conditions are to be found in temperate deciduous woodlands with patches of dense vegetation and little air movement. Additionally, *I. ricinus* may be present in appreciable numbers in regions of high rainfall on agricultural land utilized by livestock, such as rough hill land or undergrazed pastures [49]. This tick species occurs in Northern, Western, Central and Eastern regions of Europe, but is sparse in Southern Europe because of its susceptibility to desiccation. In most regions of its distribution, host-seeking activity commences in spring and early summer, with ticks being found on vegetation and animals from late March and peaking in numbers from April to July. In some areas a second, less intense, phase of questing activity occurs in the autumn, and as a result of global warming, tick activity now occurs more frequently in winter [50]. All active stages of larva, nymph and adult ticks ambush their hosts from the vegetation and, with the exception of the male, which generally does not feed, they attach to the skin with specialized mouthparts for several days, the duration depending on the tick life cycle stage.

In infected unfed ticks, babesia parasites occur in the salivary glands, but they are not infective until they have undergone development, which is initiated when the tick starts to feed, and takes about two days to complete. *Ixodes ricinus* was first shown to be the vector of *B. divergens* in transmission experiments using splenectomized calves [51]. It appears that infections are chiefly acquired by adult females while feeding on an infected host and they then pass the infection transovarially to the next generation of ticks, all stages of which (except perhaps for males) are capable of transmitting the infection [52]. While infection acquisition by immature stages has been suggested, this arose out of laboratory studies involving gerbils (*Meriones unguiculatus*) as hosts, and no direct evidence for the implied transstadial transmission that might follow exists [53]. *Ixodes ricinus* was also shown to be the likely vector of *B. venatorum* by Bonnet et al. [54], who demonstrated probable transovarial transmission from adult ticks feeding on roe deer to the next generation larvae. *Ixodes ricinus* as a vector of *B. venatorum* was validated in a subsequent in vitro study, in which both nymphs and females were shown to acquire infections and to transmit them transstadially and transovarially [55]. One of the consequences of transovarial transmission and transstadial persistence in *B. divergens* and *B. venatorum* is that theoretically infected ticks could occur in regions where infected reservoir hosts are not present, particularly as a result of the deposition of infected larvae by birds. Transstadial transmission of the third species, *B. microti*, involving acquisition by *I. ricinus* larvae from rodent hosts, followed by infection of hosts by nymphs, was reported by Walter and Weber in 1981 [56] and confirmed by Gray et al. in 2002 [57]. Additionally, the latter study showed that transovarial transmission of *B. microti* does not occur, that the parasite does not persist in the tick beyond one moult and that *I. ricinus* can transmit a zoonotic American strain, suggesting that it might be the vector of more than one European strain of the parasite.

Using PCR-based techniques, *Babesia* spp. are detectable in unfed free-living *I. ricinus* ticks. *Babesia divergens* occurs at a very low rate in ticks and is often undetectable even when the ticks have been collected from pastures where bovine babesiosis has occurred recently [58,59]. *Babesia venatorum* occurs at a slightly higher frequency [60], and as a parasite of roe deer, almost is always in ticks from woodlands. Tick infection rates of *B. microti*, also associated with rodents and woodlands, tend to be much higher, sometimes exceeding 10% [61].

Human *B. microti* babesiosis cases are exceedingly rare in Europe, despite the fact that this parasite occurs commonly in rodents and can be readily detected in unfed *I. ricinus* ticks [60], which are proven vectors of at least some Europe strains [57]. However, it should be noted that another tick species, *Ixodes trianguliceps*, is the dominant *B. microti* vector in many regions [62], and this tick species rarely bites humans. Furthermore, the parasites it transmits may not be infective for *I. ricinus*. These factors probably contribute to the low disease rate, but nevertheless, serosurveys indicate considerable exposure of the human population to infection [63].

## 5. Epidemiology

### 5.1. Autochthonous Babesiosis Cases

Human babesiosis is very rare in Europe, although the exact number of European cases is difficult to establish. Gorenflot et al. reported 22 cases of human babesiosis caused by *B. divergens* up to 1998 [64]. They occurred in France (10), the British Isles (6), Russia (1), Spain (2), Sweden (1), Switzerland (1) and present-day Croatia (1). Some cases were not published but communicated personally, and not all of them were confirmed using molecular methods [1,46,47,64,65,66,67,68]. From 1998 until the present, at least 13 additional cases were published in France [69,70,71,72], Portugal [14], Norway [73], Spain [74,75,76], Turkey [77], Finland [44], Ireland [78] and the UK [79] (Table 1). Overall, this amounts to more than 50 cases, with 35 attributed to *B. divergens*, 5 to *B. venatorum* and 11 to *B. microti* (excluding imported cases) (Table 1, Table 2, Table 3).

Despite the rarity of the disease in Europe, several serosurveys suggest that infections may be surprisingly frequent. For example, Hunfeld et al. reported positive values of 5.4% and 3.6% for *B. divergens* and *B. microti*, respectively, in a sample of 467 sera collected from the general population in Germany [63], and in Slovenia, IgG titers in 215 samples ranged from 8.4% to 2.8% depending on the IFAT cut-off [95]. However, in contrast to the USA, where transfusion-transmitted infections occur quite frequently, to date, only a single case in Europe (*B. microti* [8]) appears to have resulted from a blood transfusion. At the present time, therefore, and despite the increasing evidence for mild and asymptomatic infections [22,71,72] and the relative frequency of blood transfusions in the population, this form of transmission appears to carry a low risk of babesiosis in Europe.

The two greatest risk factors for zoonotic European babesiosis are exposure to *I. ricinus* ticks (though patients may not be aware of a tick bite) and splenectomy (Table 1 and Table 2). Even in the few spleen-intact cases there was usually evidence of splenic dysfunction or other immune incompetence, as discussed below (Section 6.1). In contrast, the few European *B. microti* cases have all been spleen-intact cases. This is also a common feature in the many cases of infection with this parasite in the USA [96]. Considering the numbers of splenectomized individuals in the European population (several hundred thousand in France alone in 1983 [64]), and the abundance of *I. ricinus* throughout Europe, the frequency of babesiosis is surprisingly low, indicating perhaps that additional immunosuppressive conditions are contributing factors in disease occurrence (Table 1), but the low infection rates of ticks, even in habitats frequented by the relevant reservoir hosts, may also be a factor in the rarity of the disease.

Another obvious risk factor for *B. divergens* infection is association with cattle, as indicated by the genetic similarity between cattle isolates and those from human cases where the parasites were sequenced (Figure 2). Although *B. divergens* has been reported in red deer, the parasite genotypes differ from those isolated from cattle or humans (Figure 1, [15]). However, this topic requires further investigation, as discussed in the previous section. Since roe deer have been identified as reservoir hosts of *B. venatorum* [55], it is reasonable to suppose that exposure to ticks in woodland is a risk factor, as discussed in Section 4.

### 5.2. Imported Babesiosis

To date, 13 cases of human babesiosis have been documented as imported to European countries. All cases were attributed to *B. microti* and were diagnosed in Switzerland [83], the Czech Republic [84], Austria [85], France [87,92], Germany [86], Poland [82,88], Spain [90,91,94], Denmark [89] and the UK [93], having been acquired in the Americas (Table 3).

### 5.3. Ambiguous Babesiosis Cases

A small number of human babesiosis cases documented from Europe do not fit into any of the categories described above. These include three symptomatic cases with unidentified *Babesia* species reported in France [64,71] and Spain [64]. Additionally, one case of coinfection with *Babesia* spp. and *Borrelia* spp. was reported in Poland. Published sequences showed 98.99% homology to both *B. divergens* and *B. venatorum*, so that exact speciation was not possible [97]. In four more cases of babesia infection, which were detected in a retrospective study in immunocompetent patients in France, species identification was not possible [72]. Finally, one *B. microti* infection, diagnosed in Spain in a 43-year-old woman with an intact spleen, was associated with moderate and prolonged disease, originally diagnosed as malaria. Over an 8-month period, she received six consecutive diagnoses of malaria with different treatment regimens that led to no clear improvement. Because all antimalarial therapies failed, the patient’s case was re-evaluated, diagnosed and eventually treated appropriately. It could not be established whether the patient acquired the infection in Europe (autochthonous) or in Equatorial Guinea (imported) [25].

### 5.4. Reports of Possible Cases with Diagnostic Deficiencies/Lack of Clarity

Of the 22 human cases of *B. divergens* infections that were documented before 1998 and described by Gorenflot et al. [64], and not all fulfill present day diagnostic standards, as some were only diagnosed microscopically without PCR confirmation, sequence analysis or serological testing [1,46,47,65,66,67,68,98,99]. Two cases reported as *B. bovis* infections were diagnosed on the basis of their appearance under the microscope only, rendering their identity questionable [1,100]. Even after 1998, at least 26 more cases were published as human babesiosis, despite diagnostic deficiencies or with lack of clarity, including 2 case reports of *B. microti* from Russia [101], 1 case from Spain [102] and 10 cases of unknown *Babesia* spp. from Montenegro [103], which were only diagnosed microscopically. Another case of *B. microti* infection in Switzerland presented doubtful microscopy, negative PCR and borderline serology [104]. A retrospective analysis of cases of babesiosis admitted to Spanish hospitals through data recorded in the minimum basic data set at discharge (MBDS) during the period 2004–2013 found 10 patients diagnosed with human babesiosis [105]. Only two of these were unequivocally identified as *B. divergens* and published [74,75]. Additionally, in a few cases, the co-infection of *Babesia* spp. with other tick-borne pathogens was reported, but unfortunately diagnoses in these cases were only performed on the basis of clinical presentation [105] or had deficiencies in the diagnosis of babesiosis [104,106]. For example, in a case of septic babesiosis reported from Spain [102], the patient presented a widespread exanthema with the presence of well-established annular lesions. Biopsy of one of the annular lesions showed changes compatible with a necrolytic migratory erythema. The patient had clinical symptoms of sepsis, but the diagnosis of human babesiosis was only based on apparent positive microscopy. There are no other reports of babesia infections causing erythema figuratum, and other differential causes have to be considered for this patient such as pancreatic neuroendocrine tumor-like glucagonoma, liver diseases and zinc deficiency [107,108,109]. Similarly, Strizova et al. reported a case of a 36-year-old man in the Czech Republic who experienced severe polytrauma requiring repetitive blood transfusions. Six months later he presented with possible Reiter’s syndrome consisting of arthritis, conjunctivitis and urethritis. The diagnosis of human babesiosis caused by *B. microti* mimicking Reiter’s syndrome was performed only based on apparent positive microscopy and the lymphocyte transformation test, which has not been evaluated for its sensitivity and specificity in the diagnosis of babesiosis [110]. Again, it is important to stress that the parasite is difficult to identify using microscopy alone, particularly if parasitemia is low, and confusion with platelets of staining artefacts is common (further discussed in Section 6).

## 6. Clinical Course of Infections

### 6.1. Pre-Disposing Factors of Acute Disease

General pre-disposing factors associated with a higher risk of symptomatic human babesia infection and more severe illness are splenectomy, impaired cellular and/or humoral immunity and advanced age [31,32,111]. The latter is explained by the decline in cellular immunity in patients over the age of 50 years [37].

In Europe, most severe cases were either splenectomized [1,4,5,6,7,14,45,46,47,70,73,74,77,86], or they had a rudimentary spleen and hyposplenism [44,78]. Immunosuppressive co-morbidities associated with severe babesiosis are hematological malignancies such as Hodgkin’s disease [4,5,45], B-cell lymphoma [4], acute myeloid leukemia [8], hairy cell leukemia [6], T-cell lymphoma [7] and HIV [74]. Patients with these malignant diagnoses and moderate-to-severe babesiosis were often on chemotherapeutic drugs with additional immunocompromising effects including prednisone, doxorubicin, cyclophosphamide, methotrexate, bleomycin and rituximab [4,5,6,7,8]. In human infections of *B. microti* in the United States, it has been reported that the severity of the disease increased with increasing parasitemia [112], with severe outcomes or complications of babesiosis associated with parasitemias of >4% [113] or >10% [114,115]. A few exceptions include reports of death in babesiosis patients with parasitemia <3% [116]. This latter observation has also been made in critically ill patients in Europe [76], but in most European cases, parasitemias in patients with complications ranged from 10% up to 80% in *B. divergens* infection [14,44,46,70,75,77,78,79], 4% to 30% in *B. venatorum* infection [4,5] and 3% to 20% in *B. microti* infection [90,91,92,93].

One of the very rare but potentially fatal complications of babesiosis is hemophagocytic lymphohistiocytosis (HLH) [87,117,118,119,120,121]. In HLH, normal downregulation of activated macrophages and lymphocytes does not occur, resulting in excessive inflammation, hypercytokinemia, abnormal immune activation and tissue destruction. Dysregulation is due to the inability of natural killer cells and cytotoxic lymphocytes to eliminate activated macrophages. HLH is classified into a genetically determined primary form and a secondary form that occurs in older people with underlying conditions such as infections, malignancies and autoimmune disorders [122]. In Europe, HLH has been reported in at least four patients with babesiosis caused by *B. divergens* [74,123], *B. venatorum* [7] and *B. microti* [87]. Cofactors for severe disease in these patients were older age [87], and a newly diagnosed HIV infection in one patient [74].

In many European cases, detailed laboratory parameters are frequently not available, so that a retrospective analysis of potential clinical factors that may have rendered patients to be more susceptible is not feasible. Future reports on European cases should consider risk factors for severe disease that have been reported for *B. microti* infection including hemoglobin level <10 g/dL, parasitemia ≥10 days, elevated alkaline phosphatase >125 U/L, total white blood cell count >5 × 10^9^/L and prior existing cardiac abnormalities [113,114].

### 6.2. Babesia divergens

This section describes the clinical course of babesiosis cases reported over the last 21 years (2000–2021) in asplenic, hyposplenic and normosplenic patients, who presented with mild-to-severe disease, dependent on age, immune status and co-morbidities (Table 1).

#### 6.2.1. Features of the Disease in Asplenic and Hyposplenic Patients

Since 2000, six patients who had been immunocompromised by splenectomy developed severe infections [14,69,70,73,74,77]. Two other patients had rudimentary spleens probably resulting in functional aspleny [44,78]. The period from onset of symptoms to the diagnosis of babesiosis ranged from one month prior to admission to four days post admission. Before obtaining a correct diagnosis of human babesiosis, patients were misdiagnosed with malaria, fever of unknown origin and *Mycobacterium* spp. infection secondary to HIV. Frequently presented symptoms were a fever up to 40 °C, headache, abdominal and back pain, fatigue, hemolysis with or without anemia and jaundice. Severely ill patients developed acute renal failure, hemophagocytic syndrome, atrial fibrillation, ARDS, hospital-acquired pneumonia, pulmonary aspergillosis, septic shock and multiorgan failure [14,44,69,70,73,74,77,78]. Parasitemias ranged from 1% [69] to 60% [70]. Only two of these eight patients (25.0%), a 66-year-old man [14] and a 53-year-old man [44], succumbed to the infection indicating a significant improvement in survival rates compared to cases reported before 1998 [64].

Interestingly, the two patients who were not splenectomized but were hyposplenic developed very severe disease. One case involved a 79-year-old man in Ireland with a 5-day history of fever, malaise, nausea, generalized pains and dark-colored urine. The patient remembered removing a tick from his arm two weeks prior to the onset of illness. He had been diagnosed with celiac disease several years before admission. A peripheral blood smear revealed babesiosis with 20% parasitemia and the presence of Howell–Jolly bodies. The patient received antibabesial treatment but developed several complications including acute renal failure, ARDS and hospital-acquired pneumonia. Altogether he was hospitalized for 61 days. An MRI scan later revealed an atrophic spleen [78]. Defective splenic function affects more than one-third of adult patients with celiac disease [124]. Eliminating gluten from the diet may improve splenic function [125], but this works inconsistently and apparently not in those patients who have already developed splenic atrophy [125,126].

The second hyposplenic patient was a 53-year-old man from Finland who succumbed to the infection. He showed typical symptoms, but also had dark streaks on his arms and legs, probably caused by massive intravascular hemolysis, and an erythema migrans indicating possible co-infection with *Borrelia burgdorferi* s.l. On post-mortem examination, splenic atrophy was found, probably caused by alcohol consumption and/or by a previous history of alcohol-induced pancreatitis. Unfortunately, no mention was made of any investigation for other possible causes of this case of hyposplenism, such as celiac disease or other autoimmune diseases [44].

#### 6.2.2. Features of the Disease in Normosplenic Patients

*Babesia divergens* parasitemias in immunocompetent individuals are generally lower than in immunocompromised patients and are often difficult to detect [71]), but at least six cases of infection have been reported in normosplenic patients during the last 21 years [71,72,75,76,79]. The course of disease ranged from mild [71] to severe [75,79] and even lethal [76], and parasitemias ranged from 0.29% [71] to 20% [78,79]. Mild cases presented with fever, chills, headache, arthromyalgia, leukopenia and elevated liver enzymes [71,72]. More severely ill patients had fever, malaise, vomiting, abdominal pain, hemolytic anemia, jaundice, hemoglobinuria and acute renal failure [75,76]. One of these six patients (16.7%) died [76].

One of the cases involved a 46-year-old forest ranger in Spain who was hospitalized after 3 days of fever, severe abdominal pain, jaundice and black and red deposits in his urine. Laboratory parameters indicated hemolytic anemia. CD4+ T cell counts were normal and serologic tests and blood cultures for hepatitis and HIV, as well as *Bartonella*, *Brucella*, *Leishmania*, *Leptospira* and *Borrelia* spp. were negative. Initial parasitemia was 10%, diminished gradually and resolved 10 days after starting a 12-day course of antibabesial therapy of quinine and clindamycin. Interestingly, hemolytic anemia remained severe, as evidenced by low hemoglobin. The patient’s illness unexpectedly relapsed on day 18 after treatment. Parasites were again detected in blood samples and he was put on a 7-week course of combined atovaquone/proguanil and azithromycin [75].

In another case, a 72-year-old immunocompetent patient in the UK developed a parasitemia up to 20%. Unfortunately, we have no information about the clinical course of the disease in this patient. Older age was the only known risk factor [79]. Old age was probably also a factor in a fatal *B. divergens* infection in an 87-year-old woman from Spain who was hospitalized after three months of low-grade fever, malaise, vomiting, decreased appetite, jaundice and hemoglobinuria. In her case, parasitemia was low (2.9%). Although she received effective antibabesial treatment that cleared the parasites by day 15 following admission, she developed acute renal failure, nose and mouth bleeding and extensive cutaneous hematomas as result of disseminated intravascular coagulation, which resulted in death [76]. In addition to her advanced age, the patient also had complex cardiovascular co-morbidities, which in *B. microti* infections have been identified as risk factors for severe disease [113].

The first indication that *B. divergens* may cause relatively mild infections was reported by Martinot et al. in 2011 in France [72]. They detected intraerythrocytic parasites and *B. divergens* DNA in a 37-year-old woman with an unremarkable medical history, who presented with fever, headache and arthromyalgia two weeks after a tick bite and who recovered without specific antibabesial medication. Infected erythrocytes were also observed in a 35-year-old man showing similar symptoms, who also recovered uneventfully, but it was not possible to speciate this parasite using PCR. More recently (2018), also in France, Paleau and others detected *Babesia* spp. infection in six patients with flu-like symptoms, using a combination of tests that included PCR [72]. *Babesia divergens* was definitively identified in two of the cases. Interestingly, one patient was additionally diagnosed with *K. pneumonia* septicemia and hepatic abscesses, perhaps indicating an unrelated co-infection or a superinfection of acute or chronic human babesiosis. Another patient was diagnosed additionally with hemolytic anemia and acute pneumonia. Although pulmonary symptoms have been described in relation to human babesiosis, an unrelated co-infection could not be ruled out. Finally, babesiosis was diagnosed in a patient presenting with febrile eosinophilic panniculitis, which is an unusual cutaneous symptom in babesiosis. Unfortunately, there is no information on the patient’s history, medication or co-morbidities [72].

### 6.3. Babesia venatorum

Altogether, five cases of *B. venatorum* have been described in Europe to date, in Austria [4,6], Italy [4], Germany [5] and Sweden [7] (Table 2). An additional unpublished case from Poland, listed in GenBank under accession number KP072001, is not discussed in this section. Interestingly, the five patients were over 50 years of age, splenectomized and diagnosed with hematological malignancies including Hodgkin’s disease [4,5,7] and hairy cell leukemia in the fifth [6]. One of the Hodgkin’s patients also had large B-cell lymphoma [4]. Four patients received immunosuppressive drugs including bleomycin [4], prednisolone + rituximab [5], methotrexate [6] and cyclosporine + prednisolone [5]. One patient developed mild [4], one patient mild-to-moderate [6] and three patients moderate-to-severe [4,5,7] disease. Reported symptoms were recurrent episodes of fever, progressive weakness, shortness of breath, thrombocytopenia, jaundice, abdominal pain, hemolytic anemia with elevated serum lactate dehydrogenase, elevated indirect bilirubin values, low haptoglobin levels and acute renal failure with dark urine as result of hemoglobinuria [4,5,6,7]. In two patients, a positive direct Coombs test led to an initial misdiagnosis of autoimmune hemolytic anemia potentially due to ongoing Hodgkin’s disease [5] or ongoing hairy cell leukemia [6]. Moreover, elevated C-reactive protein and procalcitonin levels suggested persistent infection in two patients [5,7]. Bone marrow examination of the Swedish patients showed a few phagocytosing macrophages and monocytosis leading to a tentative diagnosis of hemophagocytic lymphohistiocytosis with supporting laboratory evidence including elevated triglycerides, ferritin and soluble interleukin-2- receptor [7]. Parasitemias in the *B. venatorum* infections ranged between 1.3% [4] and 30% [4,6]. While all patients eventually seroconverted [4,5,6,7], the German case remained seronegative for specific antibodies for several months and suffered a relapse after the conclusion of the initial treatment. Moreover, retreatment with atovaquone and azithromyin for two months was unsuccessful in clearing the parasite, and low-level parasitemia persisted for several months despite maintenance therapy with atovaquone, possibly due to the previous combined application of rituximab and prednisolone, which have highly immunosuppressive effects. The Swedish patient also had fluctuating parasitemia for several months, although it was not clear whether this was a natural feature of the infection or due to injections with human immunoglobulin [7]. All five patients were cured [4,5,6,7]. A study in China on people who sought medical help after a tick bite detected 48 out of 2912 individuals with *B. venatorum* infections [127], suggesting that cases caused by this parasite generally take a milder course than those caused by *B. divergens*, requiring special awareness for detection and appropriate treatment.

### 6.4. Babesia microti

#### 6.4.1. Autochthonous *B. microti* Infections

*Babesia microti* infections in humans are rarely reported outside the United States. So far, only 11 autochthonous cases have been reported from Europe (Table 3). A marked characteristic of *B. microti* infections, in contrast to those caused by *B. divergens* and *B. venatorum*, is that the vast majority of cases have occurred in normosplenic patients. Moreover, asymptomatic infections appear to be common. However, clinical manifestations in asplenic patients are very similar to those caused by *B. divergens* and *B. venatorum*, often fulminating and resulting in death [32,42,113,114,128,129]. Patients who have recovered from acute babesiosis often maintain persistent asymptomatic parasitemia lasting for several months. In immunocompromised individuals, *B. microti* infections may even persist through multiple courses of treatment [42,130]. Relapse of illness is also more common in immunocompromised than previously healthy adults, but even in this group it may occur as long as 27 months after the initial illness [131,132]. Since many infections are asymptomatic and/or persistent, transmission of *B. microti* through blood transfusion is a serious public health threat in the USA [32,133]. Transfusion-related transmission may arise at any time of the year and incubation periods can be much longer than in tick-transmitted infection [134,135].

The first reported European case of *B. microti* occurred in Belgium in an otherwise healthy man in his 40s in Belgium in 1981 [81]. He suffered from fever and weight loss of 8 kg within one month. His serum was reactive for *R. conori* and *B. microti* and *B. rodhaini*. The patient was cured, but it is not clear whether he was infected with *B. microti* or if antibodies showed cross-reactivity with *Rickettsia* spp. [81]. The first validated case occurred in Germany [8] and is the only one so far in which parasites were observed within erythrocytes. The other nine documented cases occurred within the last six years in Poland [22,24] and Spain [25] and were diagnosed by the detection of parasite DNA. All patients had an intact spleen, and in all but one patient immunocompetence could be assumed. The exception was the German case, who was immunocompromised because of treatment for myeloid leukemia. In this case, a moderate disease developed with fever, heavy chest pain, hypertension, tachycardia and pancytopenia. Microscopy showed an initial parasitemia of 4.5% [8]. However, it is difficult to determine whether pancytopenia resulted from the *B. microti* infection, the underlying disease of acute myeloid leukemia or a combination of both. It is notable that this patient showed acute onset of babesiosis with clinical symptoms of coronary heart disease, probably due to ongoing anemia. Acute disease manifestation was followed by subsequent seroconversion for *B. microti*-specific antibodies six weeks later and points to a newly acquired infection rather than an acute exacerbation of a pre-existing subclinical parasitemia. The specific antibody response disappeared four weeks after seroconversion, probably owing to the start of another cycle of chemotherapy with cytarabine and idarubicin. The source of infection in this case was apparently an infected blood transfusion from an asymptomatic blood donor [8], whereas in the other patients tick-bite transmission is probable. The other patients with *B. microti* infections included two individuals, who were randomly identified as part of a study of forestry workers, employed in the Podlaskie province of Eastern Poland. Both were >45-years-old adults and reported several tick bites while working in forests over the preceding two years [22]. Six patients in Poland and one patient in Spain had a mild disease with nonspecific clinical symptoms such as fever, muscle pain, joint pain, headache, vertigo, fatigue and general malaise [24,25]. The case in Spain is an example of low-grade chronic human babesiosis caused by *B. microti*, with intermittent symptoms for a period of at least four months. Such cases may go undiagnosed in immunocompetent patients [25]. Altogether, four patients seroconverted and all those with symptoms were cured.

#### 6.4.2. Imported *B. microti* Infections

Parasitemias of imported cases ranged up to 20% [90,91,93] (Table 3). Clinical symptoms were similar to those of autochthonous cases characterized by fever, fatigue, malaise, chills and headache, as well as signs of hemolytic anemia, thrombocytopenia, acute renal failure and multiorgan failure in severe cases [90,91,93]. Unusual symptoms were neck stiffness in a patient with additionally diagnosed neuroborreliosis [88], and lower back pain, continuous knee pain and erythematous skin changes without any detected co-infection [89]. Bone marrow aspiration of a patient with severe pancytopenia showed typical hemophagocytosis [87]. Although all 13 patients with imported *B. microti* infections were evidently in good health for travel [82,83,84,85,86,87,88,89,90,91,92,93,94], an 83-year-old man diagnosed with low-grade lymphoplasmacytic lymphoma died of the infection [93]. *Babesia microti* infection should definitely be a differential diagnosis in Europe, especially for patients with a travel history to the Americas.

## 7. Laboratory Diagnostics

As human babesiosis can take a fulminant course of disease, especially in immunocompromised patients infected with *B. divergens*, rapid diagnosis is essential. A study of patients infected with *B. microti* reported that cases where diagnosis was delayed for 7 days or more were significantly associated with more severe disease [115]. In Europe, misdiagnoses (malaria, autoimmune hemolytic anemia with positive Coombs test) and lack of awareness of the existence of *Babesia* spp. as a causative infective agent have occasionally led to delayed diagnosis in the past, resulting in prolonged and potentially life-threatening disease [1,14,25,68,85] (Table 1, Table 2, Table 3). Indeed, in some cases, human babesiosis was only diagnosed post mortem [1,68]. We strongly recommend that diagnostic procedures for babesiosis should be initiated in patients that present with intermittent fever, fever of unknown origin or signs of hemolytic anemia. Patient records should include information on potential immunocompromising conditions, exposure to ticks, having received blood transfusions and travel to the USA or China within the last 6 months.

Clinical laboratory diagnosis of human babesiosis is challenging and it is uncertain whether automated hematology analyzers can reliably detect piroplasms. Where there are typical clinical symptoms, a positive Coombs test in combination with hemolytic anemia and elevated procalcitonine levels is highly suggestive of babesiosis and should prompt further diagnostic testing [5,136].

### 7.1. Light Microscopy

Ideally, direct pathogen detection is recommended for a definitive diagnosis. The gold standard is microscopic detection in a Giemsa or Romanowsky stained blood smears [111,137]. However, early in the course of infection or because of a low-level parasitemia, parasites may be difficult to find and smears from serial blood collection must be investigated [80,137,138]. Malaria is the most important differential diagnosis because the early stages of *Plasmodium* spp. intraerythrocytic ring forms lack the parasite pigment (hemozoin) that occurs in later stages, and thus resemble the round forms of *Babesia* spp. Hence, reliable *Babesia* spp. identification is not possible microscopically unless paired pyriforms or tetrads (Maltese crosses) are seen [111]. Piroplasms appearing in thin blood smears are ring- or pear-shaped forms with reddish chromatin and slightly bluish cytoplasm (Figure 1). Babesia merozoites arranged as tetrads usually occur in cases where there is a high parasitemia and are mainly observed in Clade 1 *Babesia* spp. (*B. microti*, *B. duncani*), but also in *B. divergens*. Parasitemias can range from <1% to 80% of infected erythrocytes and are mostly low in immunocompetent patients and at the onset of disease. Therefore, a thorough evaluation of ≥300 fields of vision and serial preparation of multiple smears is recommended [111,137]. It is important to stress that for species identification, microscopical detection of parasites in blood smears without additional molecular analysis of the pathogen is not sufficient.

### 7.2. Molecular Diagnostics

Nucleic acid testing is usually performed as a PCR targeting the 18S rRNA gene. This test is sensitive and specific in detecting *Babesia* spp. from clotted or EDTA blood. Sequencing of the 18S rRNA gene can be used for species identification, which has an epidemiological and therapeutic significance. The detection limit is approx. 1–3 parasites/μL of blood, and thus below that of microscopic methods [139]. There are various modifications of the test format and the molecular target structure including DNA/RNA hybridization (e.g., FISH), and real-time PCR methods [4,139], but there is currently no commercial test or sufficiently validated protocol available in Europe for diagnosis confirmation by a broadly accepted gold standard test [111].

### 7.3. Culture

*Babesia divergens*, *B. microti* and *B. duncani* can be cultivated in gerbils, mice and hamsters, respectively, while *B. venatorum* has not yet been adapted to a laboratory animal species. Approximately 0.5–1 mL of EDTA or heparin anticoagulated whole blood are inoculated intraperitoneally, and the animal blood is monitored at least once a week for up to two months. Parasitemia is detectable after one week at the earliest but can be reliably detected after up to four weeks. There are many reasons why xenodiagnosis is impracticable in routine laboratories (e.g., labor-intensive and time-consuming process, availability, ethics and sensitivity). Likewise, the in vitro cultivation of piroplasms, which is possible in principle, requires sophisticated techniques, and is thus labor intensive and costly. Having in mind these practical drawbacks, culturing is reserved for specialized laboratories, although a broader approach to cultivating more isolates both from the veterinarian and the human medical fields is clearly desirable [111,140].

### 7.4. Infection Serology

The indirect immunofluorescence assay is the most commonly used serological test method. Cut-off titers for IgG antibodies from 1:32 to 1:160 were found to be sensitive (>88%) and specific (>90%) in multicenter studies with *B. microti* and *B. divergens* antigens [63,141]. However, cut-off titers should be adjusted to the local seroepidemiological situation and circulating *Babesia* species [63]. IgG titers of ≥1:1028 occur during the course of infection, which then decrease to titers of 1:64 within months to years. IgG assays do not reliably differentiate between acute, chronic or past infections [63,111,136,141]. On the other hand, IgM antibodies are detectable from approx. two weeks after the onset of symptoms onwards and indicate acute infection [141,142]. However, since false IgM-positive test results are common, particularly as part of untargeted testing in non-endemic areas, a two-step procedure is required in which only IgG-positive samples are further tested for the presence of IgM antibodies [63,140]. Assays that detect anti-*B. microti* antibodies do not detect antibodies against *B. duncani*, *B. divergens* or *B. venatorum* [143]. In contrast, cross-reactivity between *B. divergens* and *B. venatorum* can be exploited diagnostically [5,137].

In addition to the general limitations of immunofluorescence assays (unknown test quality, investigator dependent variability, etc.), false-positive reactions have been described in sera from rheumatic patients and from patients with other, especially closely related infectious diseases such as malaria and toxoplasmosis [63,143]. Furthermore, the antibody response may not yet be present in the early phase as shown in acute European case reports or may be absent in immunocompromised individuals [5,136,140]. Therefore, it is not suitable for acute diagnosis but primarily for epidemiological purposes. Several publications describe other immunoassay formats (e.g., enzyme immunoassays, bead-based assays or immunoblots) that use a wide variety of antigens [140]. However, standardized serological test methods that have been validated by multicenter studies are currently not available in Europe due to low demand and lack of diagnostic evaluation.

Finally, it should be stated that, except for research and surveillance purposes, the practice of generally applying multiplex approaches for molecular diagnostics and/or serology in patients after a tick bite or in individuals with suspected Lyme borreliosis is not recommended, because from a statistical stand point, such diagnostic regimes will end up with many false-positive test results given the generally low incidence of tick-borne infections other than Lyme borreliosis in most European countries.

## 8. Clinical Management

Several drugs are available for the treatment of human babesiosis (Table 4), but their efficacy is variable, particularly against *B. microti*, which animal studies suggest is less susceptible to classic antibabesials than are *B. divergens* and *B. venatorum* [144]. However, available information on antibabesial susceptibility from case reports and clinical investigations suggests that there is no convincing scientific evidence for any clinically relevant differences in the susceptibilities of the pathogenic *Babesia* spp. to the therapeutic agents commonly used to treat human babesiosis [111,145]. Nevertheless, there is room for improvement in drug efficacy, particularly in relation to side effects, drug resistance and speed of response. In the case of most infections caused by *B. divergens* and *B. venatorum*, as well as severe cases of *B. microti* infection, the speed of response to antibabesial administration is particularly important, and adjunct measures are often necessary. Most of the recent cases of human babesiosis caused by previously unknown *Babesia* spp. have responded to antibabesials used against known species [111,145]. However, until further data become available, treatment of infections caused by unknown *Babesia* spp. should include close monitoring of the course of parasitemia and long-term follow-up of such patients.

### 8.1. Babesia divergens

Although sporadically observed in immunocompetent patients with viral-like illnesses, clinical cases of *B. divergens* have almost always been reported in asplenic or spleen-impaired individuals [71,78,111]. Many *B. divergens* infections in the past ended fatally with general organ failure occurring four to seven days after the initial presentation of hemoglobinuria. Outcome data in severely ill asplenic individuals show a mortality rate of 42% [27,70,71,137,146]. Consequently, the status of asplenic *B. divergens*-infected patients is regarded as a medical emergency, requiring immediate treatment to arrest hemolysis and prevent complications [111,137]. The combination of clindamycin and quinine for 7 to 10 days (Table 5) dramatically improves disease outcome [137,146,147,148,149], but in recent years, a more favorable disease course has been increasingly reported for *B. divergens*-infected patients, including those not treated with a full course of clindamycin and quinine because of quinine side effects [70,111,150]. These findings underscore the impact of improved adjunctive measures provided by modern intensive care medicine, including exchange transfusion [111,137]. This measure is usually reserved only for the most extremely ill *B. microti*-infected patients but has also been recommended for all severe *B. divergens* cases [27,111,137,145]. Alternative treatment options for *B. divergens* infections have included clindamycin monotherapy or imidocarb in conjunction with the above-mentioned adjunctive measures (Table 4) [69,102,111,137,151]. Imidocarb, one of the most effective antimicrobials for use in *Babesia*-infected animals, is highly active against this organism in vitro [152]. It was used successfully to treat two Irish patients infected with *B. divergens* but is not licensed for use in humans [153]. Atovaquone proved more effective than imidocarb in an experimental *B. divergens* gerbil model and perhaps should be considered in combination with azithromycin for treatment of *B. divergens* infections and more generally for those caused by any *Babesia* s.s. species [152]. Atovaquone, together with either azithromycin or proguanil, has been used in three recent cases, following problems with toxicity or inadequate efficacy of other drug regimens [74,75,76], and it resulted in patient recovery in two of them [74,75].

Although quinine, clindamycin, atovaquone and azithromycin, and some in combination, are proven antibabesials for the treatment of *B. divergens* infections in humans, there are concerns about rapid efficacy, drug resistance and recrudescent infections. However, cases do not occur frequently enough to justify research in drug discovery and development for human treatment alone. In recent years a significant number of drugs have been tested against this parasite in vitro for veterinary use, for example atranorin [154], cryptolepine [155], fusidic acid [156], hydroxyurea and eflornithine [155], myrrh oil [157] trans-chalcone and chalcone 4 hydrate [158], and the hope is that promising drugs will also prove useful for human infections.

### 8.2. Babesia venatorum

In general clindamycin with or without quinine and with or without subsequent combined atovaquone and azithromycin treatment have been used successfully in European cases of *B. venatorum* infection [5,6,7]. Problems with speed of response to therapy and parasite persistence occurred in one case [5].

In contrast to the more sporadic occurrence of *B. venatorum* cases in Europe, the disease is endemic in northwestern China with more than 48 reported cases [159,160,161,162], all of which were immunocompetent, in contrast to European patients. In these cases, 4 of the 48 Chinese patients received clindamycin alone and no deaths were reported [162]. Although the clinical course of *B. venatorum* generally seems to be milder than that of *B. divergens*, clinicians should be aware that immunocompromised patients might experience relapse and persistence of infection despite antimicrobial treatment. In such cases, it is important to monitor parasitemia by blood smear examination and PCR analysis and provide long-term clinical follow-up [5,111].

### 8.3. Babesia microti

Autochthonous *B. microti* infections in Europe are rare and most cases have been reported in travelers, mainly those returning from the USA. In such cases, treatment should follow American standards [145]. Animal studies showed that regimes of azithromycin in combination with quinine [163], azithromycin with atovaquone [164] and atovaquone with clindamycin [144] were all effective (Table 4 and Table 5).

Randomized trials in humans infected with *B. microti* showed that atovaquone plus azithromycin therapy was as effective as the standard quinine/clindamycin combination and there were fewer side effects (15% versus 72%) [165]. In view of the low risk of side effects associated with atovaquone/azithromycin, it has been argued that all patients diagnosed with *B. microti* infection should be treated with this drug combination [111,137]. In severe cases, similar adjunctive measures to those used for *B. divergens* infections may be necessary [111] (Table 5).

Major obstacles to the development of new drugs against *B. microti* are, firstly, that a continuous in vitro culture system is lacking for this parasite despite much research on the topic, and secondly, that although continuous culture systems already exist for *Babesia* s.s. species such as *B. divergens* [152], antibabesials developed against these parasites appear to be relatively ineffective against *B. microti* [144]. However, the recent successful development of continuous in vitro culture systems for *Babesia duncani*, using human or hamster erythrocytes [166,167] promises progress in this area since *B. duncani* is more closely related to *B. microti* than to the *Babesia* s.s. species [10].

### 8.4. Exchange Transfusion Management

Exchange transfusion has been recommended for severe *B. microti* infection characterized by parasitemias of more than 10%, and/or severe anemia (hemoglobin <10 g/dL) and/or evidence of organ dysfunction (hepatic, pulmonary or renal compromise), as well as for all emergency cases involving *B. divergens* [111,137,145]. Such a procedure can contribute to the rapid reduction of parasitemia, correction of anemia and elimination of toxins and harmful metabolites, but it is complex and should take place under the supervision of specialised hematologists, taking into account the status and co-morbidities of the patient. Although erythrocyte exchange transfusion as an adjunct to treatment of severely ill patients can be life-saving in selected cases [168], it requires more research, since there has not yet been a prospective clinical study of outcomes of exchange transfusion combined with antimicrobial agents, compared with antimicrobial agents alone.

## 9. Conclusions

The spread of infectious diseases among people and animals is a worldwide challenge. The One Health approach provides the opportunity to systematically and comprehensively address emerging zoonoses such as human babesiosis in order to increase awareness of the risk of infection and improve precise diagnostic and seroprevalence tests and treatment protocols. Advances in laboratory methodologies are required to increase our knowledge and understanding of the diversity of zoonotic *Babesia* species and the roles that domestic animals, wildlife and tick populations play in their maintenance. Further development of laboratory tools is necessary for babesia research, including molecular characterization of *Babesia* species and in vitro culture, particularly for testing parasite susceptibility to antibabesial drugs, and the development of screening diagnostics that can be used routinely, for example for the protection of the transfusion blood supply. The improvement of patient care continues to be important, as awareness is raised among health care professionals and the provision of information on disease prevention behavior is considered by local, national and international governmental institutions.

## Figures and Tables

**Table 1:** *Babesia divergens* infections in Europe.

Year	Country	Age, Gender, (Outcome)	Course of Disease *	Co-Morbidities Compl./Unusual Features	Misdiagnosis, Time from Symptoms until Diagnosis: Prior ad./Post ad.	Parasitemia	Antibabesial Therapy	References
1957–1998 22 cases	10× in France (8 cured, 2 died) 6× British Isles (2 cured, 4 died) 1× Russia (died) 2× Spain (1 cured, 1 died) 1× Sweden (cured) 1× Switzerland (cured) ** 1× Ex-Yugoslavia (died)		mild to lethal	Co-morbidities: Hodgkin’s disease, splenectomy, hypertension, diabetes; Compl: ARF, ARDS, shock, HLH, cardio-respiratory arrest, cardiac effusion	malaria, 3 days/10 days or diagnosis post mortem	2–80%	Drugs used: QN + CLI, QN +CHQ PNT + CTM QN + CLI + PNT QN + DOX CH + CLI CH + DOX + MEF ET	[64]
**1999–2021 Asplenic and Hyposplenic Patients**								
1999	France	44, M, splenecto-mized (cured)	mild to moderate	NI	3 days/1 day	1%	QN + CLI	[69]
2003	Portugal	66, M, splenecto-mized (died)	severe to lethal	Co-morbidities: MI 1984, subtotal gastrectomy; Compl: ARDS, ARF	malaria, 1 week/4 days	30%	QN + CLI + VI	[14]
2004	Finland	53, M, rudimentary spleen (died)	severe to lethal	Co-morbidities: severe alcohol-induced pancraetitis, diabetes type 1; Compl: Septic shock, multiple organ failure, pulmonary aspergillosis, UF: ECM	1 week/2 days	10%	QN + CLI + CFX, ET	[44]
2005	France	51, M, splenecto-mized (cured)	moderate to severe	Compl: ARF, ARDS	2 days/1 day	60%	QN + CLI	[70]
2015	Norway	58, M, splenecto-mized (cured)	severe	Compl: ARF, ARDS, atrial fibrillation	FUO, 4 days/2 days	30%	QN + CLI, ET	[73]
2015	Spain	37, M, splenecto-mized (cured)	moderate to severe	Co-morbidity: newly diagnosed HIV; Compl: HLH, ARDS	*Mycobacterium* spp., 3 days post ad.	low	QN + CLI, AZM + ATQ	[74]
2015	Turkey	28, F, splenecto-mized (cured)	moderate to severe	NI	malaria, 1 month/2 days	50%	ET, QN + CLI	[77]
2017	Ireland	79, M, hypo-splenism (cured)	moderate to severe	Co-morbidities: adult celiac disease, pulmonary TB; Compl: ARF, HAP	7 days/2 days	20%	ATQ + AZM, CLI + QN	[78]
**1999–2021 Normosplenic Patients**								
2011	France	37, F (cured)	mild	NI	TBD, 3 weeks post ad.	0.29%	DOX	[71]
2011	Spain	46, M (cured)	moderate to severe	Compl: ARF, relapse	3 days/1 day	10%	QN + CLI; relapse: AZM + AP	[75]
2018	Spain	87, F (died)	severe to lethal	Co-morbidities: ovarian tumor, malignant hypertension, transient ischemic attacks, osteoporosis; Compl: ARF, bleeding disorders, cardio-respiratory arrest	3 months/4 days	2.9%	AZM + AP	[76]
2020	France	6 patients, no information about sex and age (cured)	mild to moderate	UF/Compl: 1× unusual cutaneous symptom, 1× K. pneumonia septicemia and hepatic abscesses, 1× acute pneumonia, 1× febrile eosinophilic panniculitis	retrospective analysis	In 2/6 pos.	2 patients: DOX 2 patients: AZM + ATQ 1 patient: CTX + SPI 1 patient: COX + AMC + OFX	[72]
2021	UK	72, F (NI)	moderate to severe	NI	3 days/1 day	20%	NI	[79]

M—male, F—female, ND—not done, NI—no information, pos.—positive, ad.—admission, UF—unusual feature, Compl.—complications, FUO—fever of unknown origin, MI—myocardial infarction, TB—tuberculosis, TBD—bacterial tick-borne disease, ARF—acute renal failure, ARDS—acute respiratory distress syndrome, HAP—hospital-acquired pneumonia, HLH—hemophagocytic lymphohistiocytosis. * Classification of disease severity followed criteria suggested by Vannier and Krause (2009) [80]. ** infection probably acquired in Wales. AZM—azithromycin, ATQ—atovaquone, AP—atovaquone/proguanil, QN—quinine, CLI—clindamycin, VI—vibramycin, CTX—ceftriaxone, COX—cefotaxime, AMC—amikacin, OFX—olfloxacin, CHQ—chloroquine, PNT—pentamidine, CTM—cotrimoxazole, CH—chinin, MEF—mefloquine, ET—exchange transfusio

**Table 2:** *Babesia venatorum* infections in Europe (all cases with clinical details were splenectomized).

Year	Country	Age, Gender, (Outcome)	Course of Disease *	Co-Morbidities, Compl.	Misdiagnosis, Time from Symptoms until Diagnosis: Prior ad./Post ad.	Parasitemia	Antibabesial Therapy	References
2003	Italy	55, M, (cured)	moderate to severe	Co-morbidities: splenectomized because of Hodgkin’s disease, recently started chemotherapy for stage IIIA diffuse large B-cell lymphoma	4 days/6 days	30%	QN + CLI	[4]
	Austria	56, M, (cured)	mild	Co-morbidities: splenectomized, Hodgkin’s disease	2 days/1 day	1.3%	CLI	
2007	Germany	63, M, (cured)	moderate to severe	Co-morbidities: splenectomized, Hodgkin’s disease, immunosuppressive treatment; Compl.: prolonged, relapse	relapse of Hodgkin’s disease, AIHA 3–4 weeks/2 days	4%	QN + CLI, CLI, relapse: AZM + ATQ, ATQ for 5 months	[5]
2011	Austria	68, M (cured)	mild to moderate	Co-morbidities: splenectomized, hairy cell leukemia, immunosuppressive treatment, granular lymphocyte leukemia; Compl.: ARF	AIHA some weeks/3–4 days	30%	QN + CLI	[6]
2015	Poland	NI	asymptomatic	NI	NI	NI	NI	GenBank: KP072001
2017	Sweden	52, M (cured)	moderate to severe	Co-morbidities: splenectomized, T-Cell Lymphoma, immunosuppressive treatment; Compl.: HLH	Hemophagocytic syndrome, 2 months/2 days	4%	QN + CLI, AZM + ATQ	[7]

M—male, F—female, NI—no information, Compl.—complications, AIH—-autoimmune hemolytic anemia, ARF—acute renal failure, HLH—hemophagocytic lymphohistiocytosis, ad.—admission * Classification of disease severity followed criteria suggested by Vannier and Krause (2009) [80]. QN—quinine, CLI—clindamycin, ATQ—atovaquone, AZM—azithromycin.

**Table 3:** *Babesia microti* infections in Europe.

Year	Country	Age, Gender, (Outcome)	Course of Disease *	Co-Morbidities Compl./Unusual Features	Misdiagnosis, Time from Symptoms until Diagnosis: Prior ad./Post ad.	Parasitemia	Antibabesial Therapy	Reference
**Autochthonous *B. microti* Infections**								
1981	Belgium	In the 40 s, M, (cured)	moderate	Compl.: prolonged fever	Rickettsiosis, 1 month	NI	T, CHQ	[81]
2007	Germany	42, F, (cured)	moderate	Co-morbidities: AML, immunocompromizing treatment	MI, some weeks/10 days	4.5%	QN + CLI, AZM	[8]
2015	Poland	2 patients >45 (NI)	asymptomatic	NI	NI	ND	no treatment	[22]
2016	Poland	6 patients (cured)	mild	Co-morbidities: 1 EM, 1 TBE	NI	Neg.	no treatment	[24]
2016	Spain	35, M (cured)	mild	Compl: prolonged parasitemia	several months/few days second ad.	Neg.	ATQ + AZM, AP	[25]
**Imported *B. microti* Infections**								
1992	Poland	36, M (cured)	moderate	NI	malaria, NI	NI	CLI	[82]
2003	Switzerland	NI	mild	NI	NI	ND	NI	[83]
2003	Czech Rep.	58, M (cured)	mild	NI	28 days/some days	0.14%	QN + DOX	[84]
2010	Austria	63, M (cured)	moderate to severe	Compl.: hemodynamic shock, anuria	malaria, 2 weeks/diagnosis retrospective	high	QN + CLI	[85]
2012	Germany	38, M (cured)	moderate to severe	Co-morbidity: splenectomy after injury, Compl: pneumonia	Borreliosis, 3–4 months	8‰	QN + CLI, AZM + ATQ + DOX	[86]
2013	France	82, M (cured)	moderate	Compl: HLH	5 days/some days	3%	QN + CLI	[87]
2013	Poland	48, F (cured)	moderate	Co-morbidity: neuroborreliosis; Compl.: neck stiffness	10 days/10 days	3%	AP, DOX + AZM + CLI	[88]
2013	Denmark	64, F (cured)	moderate	Co-morbidity: RF; Compl.: erythematous skin changes	Borreliosis, malaria, IE, some days/1 week	4%	AP, AP + AZM	[89]
2015	Spain	66, F (cured)	severe	Compl.: multiorgan failure	2 days/2 days	20%	QN + CLI, ATQ + AZM, ET	[90]
2016	Spain	66, F (cured)	severe	Compl.: ARDS, ARF, multiorgan failure	malaria, 1 week/some days	20%	QN + CLI, AZM + ATQ, ET	[91]
2017	France	69, M (cured)	moderate to severe	Compl.: ARF; UF: diffuse purpura of the lower extremities	malaria, 1–2 days/few days	3%	QN, QN + CLI	[92]
2019	UK	83, M (died)	severe	Co-morbidity: LGLL; Compl.: multiorgan failure	severe sepsis, few days	>20%	Antibabesial therapy, ET	[93]
2020	Spain	72, M (cured)	mild to moderate	Co-morbidity: diabetes	15 days/1 day	0.5%	ATQ + AZM	[94]
**Autochthonous or Imported *B. microti* Infection**								
2014	Spain	43, F (cured)	moderate	Compl.: prolonged disease	malaria, 8 months	>0.5%	AZM-AP	[26]

M—male, F—female, Rep.—Republic, ND—not done, NI—no information, Neg—negative, ad.—admission, UF—unusual feature, Compl.—complications, MI—myocardial infarction, TB—tuberculosis, ARF—acute renal failure, ARDS—acute respiratory distress syndrome, AML—acute myeloid leukemia, EM—erythema migrans, TBE—tick-borne encephalitis, RF—rheumatic fever, IE—infective endocarditis, HLH—hemophagocytic lymphohistiocytosis, LGLL—low-grade lymphoplasmacytic lymphoma * Classification of disease severity followed criteria suggested by Vannier and Krause (2009) [80]. T—tetracycline, AZM—azithromycin, ATQ—atovaquone, AP—atovaquone/proguanil, QN—quinine, CLI—clindamycin, CHQ—chloroquine, ET—exchange transfusion.

**Table 4:** Commonly and experimentally used drugs for the treatment of human babesiosis (modified from Hildebrandt et al., 2013 [111]).

Drug (Generic Name)	Regular Single Dose	Application	Dosage Regimen
**Adults**	**Dose—70 kg adult**		
**Standard drugs**			
Quinine	650 mg	p.o.	3 times daily
Clindamycin	600 mg	p.o., i.v.	3 times daily
Azithromycin	500 mg/1st day, 250 mg thereafter ^a^	p.o., i.v.	once daily
Atovaquone	750 mg	p.o.	twice daily
Doxycycline	200 mg	p.o.	once daily
**Unlicensed Drugs for Human Babesiosis ^g^**			
Pentamidine	4 mg/kg/day	i.v.	once daily
Trimethoprim/sulfametoxazole	4/20 mg/kg	p.o., i.v.	twice daily
Proguanil	400 mg/day	p.o.	once daily
Imidocarb dipropionate ^h^	0.6 mg/kg	i.m.	12 hourly for 4 doses
**Children**	**Dose/kg**		
**Standard drugs**			
Quinine	8 mg ^c^	p.o.	3 times daily
Clindamycin	7–10 mg ^d^	p.o., i.v.	3 times daily
Azithromycin ^b^	10 mg/1st day 5 mg/day thereafter ^e^	p.o., i.v.	once daily
Atovaquone	20 mg/day ^f^	p.o.	twice daily

^a^ In immunocompromised patients, higher initial doses (600–1000 mg/day) may be required. ^b^ In immunocompromised patients, higher dose may be required. **^c^** maximum: 650 mg per dose. ^d^ maximum: 600 mg per dose, ^e^ maximum: 250 mg per dose. **^f^** maximum: 750 mg per dose. ^g^ In addition to standard drugs, alternative substances have been used successfully in some severe adult cases of babesiosis (see also Table 3) (111). ^h^ Imidocarb dipropionate is not licensed for use in humans. The dosing regimen for treatment of human babesiosis is derived from two successfully treated Irish cases with *B. divergens* infections (146).

**Table 5:** Commonly used drug combinations and treatment alternatives for human babesiosis with regard to parasite species and severity of the disease (adapted and modified from Hildebrandt et al., 2013 [111]).

Parasite	Mild Disease ^a^ (Drug)	Severe Disease ^a,b^ (Drug)	Adjunctive/Alternative Therapy in Severe Cases ^b^
*B. divergens*	clindamycin	clindamycin *plus* quinine	Exchange transfusion, hemodialysis *consider* atovaquone/azithromycin, atovaquone/proguanil *or* pentamidine/ trimethoprim-sulfametoxazole *as possible alternatives for severe and intractable infections*
*B. venatorum*	clindamycin	clindamycin *plus* quinine	Exchange transfusion, *Consider alternative treatment with* atovaquone/azithromycin *or* atovaquone/proguanil *in persisting babesiosis*
*B. microti*	atovaquone *plus* azithromycin	clindamycin *plus* quinine	Exchange transfusion hemodialysis *Consider adding* doxycycline *or* proguanil *in relapsing or persisting babesiosis*

^a^ Usual duration of treatment is 7–10 days. Longer treatment (>6 weeks) may be necessary in immunocompromised or relapsed patients. In immunocompromised individuals, reduction of immunosuppressive therapy may be needed if possible for clearing the parasite. ^b^ Severe illness criteria according to White et al., 1998 [113]: parasitemia > 4%, alkaline phosphatase >125 U/L and white blood cell counts >5 × 10^9^/L. Partial or complete exchange transfusion is recommended in case of high parasitemia (>10%), severe anemia (<10 g/dL) and pulmonary or hepatic failure. In severe disease cases i.v. treatment is suggested. Alternative treatments as derived from single case reports or case studies cited in the literature (Hildebrandt et al., 2013 [111]).

## Data Availability

Not applicable.
